# Amoxicillin conjugated functionalized zinc ferrite nanoparticles for enhanced antibacterial, antibiofilm, and antioxidant activities

**DOI:** 10.1038/s41598-025-08782-w

**Published:** 2025-07-10

**Authors:** Ahmed M. El-Khawaga, Karim Elmaghraby, Maurizio Orlandini

**Affiliations:** 1 Department of Basic Medical Sciences, Faculty of Medicine, Galala University, Galala City, 43511 Suez Egypt; 2 Department of Botany, Faculty of Science, Tanta University, Tanta, 31527 Egypt; 3 Department of Biotechnology, Chemistry and Pharmacy, University of Siena, 53100 Siena, Italy

**Keywords:** Nanoparticles, Amoxicillin, Antimicrobial activity, Antibiofilm, Antioxidant, Materials science, Nanoscale materials, Nanoparticles

## Abstract

To address the growing challenge of antibiotic resistance, magnetic nanoparticles were developed and characterized by providing an innovative solution. Zinc ferrite nanoparticles (ZnFe₂O₄ NPs) were synthesized using a chemical co-precipitation method, stabilized with citric acid (CA), and conjugated with amoxicillin (AX) to create ZnF-CA-AX nanocomposites. These nanoparticles were extensively characterized by their structural and optical properties. The antimicrobial activity of the nanocomposites was tested against gram-positive *Staphylococcus aureus* and gram-negative *Escherichia coli*, showing significant inhibition zones. Furthermore, the nanocomposites showed a high level of antibiofilm efficiency, and potential antioxidant activity against 1,1-diphenyl-2-picrylhydrazyl. Collectively, these findings indicate that magnetic nanoparticles can enhance antibiotic effectiveness, offering new therapeutic avenues to combat resistant bacterial infections.

## Introduction

The discovery and development of antibiotics have been pivotal in the fight against bacterial infections, marking significant milestones in medical history. However, antimicrobial resistance (AMR) refers to the capacity of disease-causing microorganisms to withstand the therapeutic actions of antibacterial drugs. Antibiotic resistance poses a serious global health threat, reducing the effectiveness of standard treatments and leading to increased morbidity, mortality, and healthcare costs. Approximately 700,000 deaths occur globally every year as a result of improper antibiotic use, which leads to resistance to traditional treatment^1^. For instance, in the United States in 2017, methicillin-resistant Staphylococcus aureus (MRSA) was connected to about 120,000 blood-borne illnesses and 20,000 fatalities^2^. Furthermore, it has been determined that carbapenem-resistant *Enterobacteriaceae* (CRE) pose a risk to public health and that immediate, intrusive measures are necessary^3^. It has been reported that antibiotic-resistant infections cause losses to the tune of $55–70 billion each year in the United States. Every year, losses in Europe exceed €1.5 billion^4^. More aggressive strains that are resistant to conventional therapies have emerged as a result of the overuse and inappropriate use of antibacterials^5^. The discovery of novel alternative ways to address this significant challenge is becoming increasingly essential due to the devastating human and economic costs associated with antibiotic resistance^6^.

Highlighting the use of nanoparticles as antibacterial delivery agents requires an understanding of how bacteria form colonies that are resistant to treatment with conventional antibiotics^7^. A developed system known as biofilm enables bacteria to endure adverse environments and establish durable colonies with a high colony-forming ability^8^. A dense and hydrated group of bacteria sticking to one surface and to one another is known as biofilm bacterial growth. It is encased in an external matrix composed of extracellular DNA, amino acids, and exo-polysaccharide (EPS)^9^. It is believed to be 1000 times more resistant to conventional antibiotic treatments than planktonic bacterial growth^10^. Numerous illnesses, such as infections of the lungs, colon, urethra, eyes, and ears, as well as infective endocarditis, gum disease, and wounds, have been connected to biofilm^11^.

The approval process for new antibacterial agents is long, costly, and complex—often takes 10 to 15 years. Combined with the rise of highly pathogenic bacteria and a lack of new antibiotic development, this has reduced the effectiveness of current treatments. To address this, researchers are exploring new strategies, such as nanomedicine, to enhance existing antibiotics. Nanomaterials can improve drug stability, enable targeted delivery, increase biofilm penetration, and reduce side effects, ultimately boosting the performance of conventional therapies^12–14^.

Recently, various metals, metal oxides, polymers, and carbon based nanoparticles (NPs) are being utilized for antibacterial remedies^15,16^. For instance, magnetic nanoparticles (MNPs) can be engineered to disrupt bacterial biofilms, enhance the delivery and controlled release of antibiotics, and improve their physicochemical properties. By addressing the limitations of conventional treatments, such as poor solubility, stability, and targeting, nanoparticles offer a promising approach to mitigate resistance AMR and enhance the efficacy of current antibiotics^17^. In this study, Amoxicillin was selected due to its broad-spectrum antibacterial activity, clinical relevance, and known limitations such as short half-life and reduced efficacy against biofilms. These challenges make it an ideal candidate for enhancement through nanotechnology. Conjugating Amoxicillin with citric acid-coated zinc ferrite nanoparticles aimed to improve its stability, biofilm penetration, and antimicrobial efficacy. Additionally, its molecular structure allows efficient binding to the nanoparticle surface, making it a suitable model antibiotic for evaluating the potential of the nanocomposite system^18^. By synthesizing zinc ferrite nanoparticles (ZnFe₂O₄ NPs), stabilized with CA and conjugated with AX, we formed ZnF-CA-AX nanocomposites, which revealed able to potentially lower required dosages and reduce side effects of the clinically used antibiotics. Table 1 listed the acronyms and abbreviations used in this study.

**Table 1:** List of acronyms and abbreviations.

Abbreviation	Definition
DPPH	1,1-Diphenyl-2-picrylhydrazyl
DMSO	Dimethyl sulfoxide
DNA	Deoxyribonucleic acid
CA	Citric acid
AX	Amoxicillin
FTIR	Fourier-transform infrared spectroscopy
XRD	X-ray diffraction
HRTEM	High-resolution transmission electron microscopy
SEM	Scanning electron microscopy
EDX	Energy-dispersive X-ray spectroscopy
ELISA	Enzyme-linked immunosorbent assay
CFU	Colony-forming unit
O.D	Optical density
MNPs	Magnetic nanoparticles
NCs	Nanocomposites
NPs	Nanoparticles
MIC	Minimum inhibitory concentration
ZOI	Zone of inhibition

## Materials and methods

### Chemicals

Iron (III) chloride hexahydrate (FeCl_3_·6H_2_O: 98%), Zinc chloride (ZnCl_2_: > 98%), sodium hydroxide (NaOH: 97%), citric acid (CA: 99%), and crystal violet (CV: > 90%) were purchased from E-Merck Products. Dimethyl sulfoxide (DMSO) was obtained from Sigma-Aldrich, and amoxicillin was acquired from Egyptian International Pharmaceutical Industries Co. Throughout the experiment, ultrapure Milli-Q water was utilized, and all chemical compounds were employed without additional purification.

### Synthesis of ZnF-CA-AX by chemical co-precipitation method

**Fig. 1:**
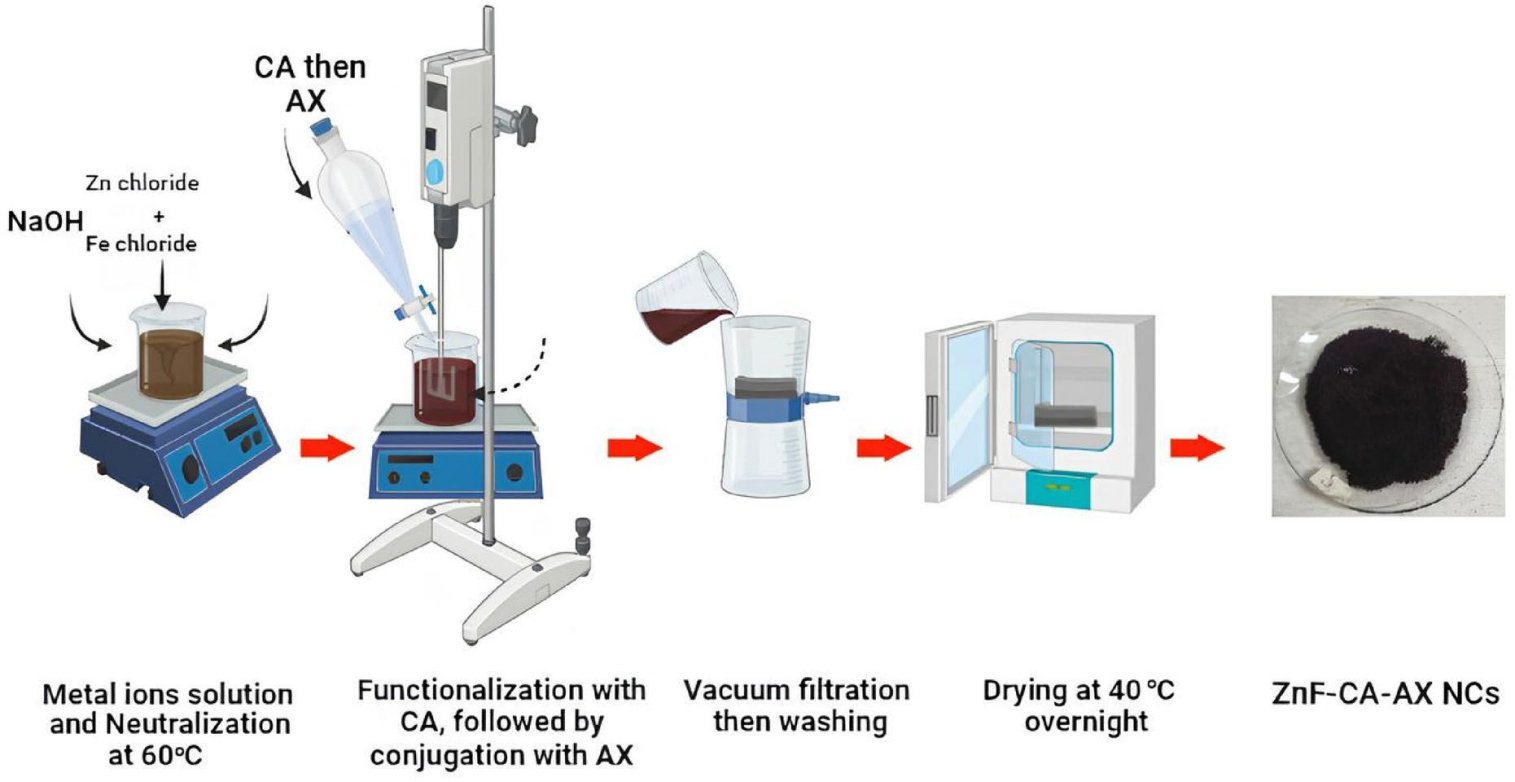
Schematic representation for the preparation of ZnF-CA-AX nanocomposite.

The most popular and efficient method for creating magnetic nanoparticles with regulated sizes and magnetic characteristics is co-precipitation. It is particularly favored in biomedical applications due to its simplicity and minimal requirement for hazardous materials and procedures^19^. In order to create magnetic nanoparticles, a base is added to aqueous salt solutions in an inert atmosphere. This can be done at room temperature or at higher temperatures^20^ as shown in (Fig. 1).

#### Synthesis of Zn Fe_2_O_4_NPs

Zinc ferrite nanoparticles (ZnFe₂O₄ NPs) are synthesized using the co-precipitation method^21^. To obtain the required composition, stoichiometric amounts of anhydrous ferric chloride (FeCl₃) and zinc chloride (ZnCl₂·6H₂O) are dissolved in distilled water. A 1 M sodium hydroxide (NaOH) solution is used for neutralization, and the reaction temperature is kept at 60 °C. After adjusting the pH of the solution to 8, the mixture is agitated for two hours. To get rid of contaminants, the precipitate is then thoroughly cleaned with distilled water. After that, the product is dried at 100 °C to remove any remaining moisture. After being evenly combined, the dried powder is sintered at 600 °C.

#### Citric acid-coated zinc ZnFe_2_O_4_ nanoparticles

The surface of the nanoparticles was stabilized with citric acid utilizing a direct addition approach to produce modified ZnFe_2_O_4_ NPs containing carboxylic groups^22^. In conclusion, ZnFe_2_O_4_ NPs were incubated in a 0.5 g/ml citric acid solution for one hour to treat them with citrate ions. After raising the reaction temperature to 90 °C, the procedure was carried out for 60 min while being constantly stirred^23^. The resulting black precipitates were gathered once the reaction mixture had cooled to room temperature. Deionized water was used to thoroughly wash the suspensions several times. The particles remained suspended under a magnetic field, demonstrating the solution’s stability. The NP dispersion was centrifuged and extensively cleaned because of the product’s excess citric acid. Citric acid-stabilized ZnFe_2_O_4_ NPs are known as CA-ZnF NPs.

#### Amoxicillin (AX) conjugation with citric acid-coated zinc ferrite (CA-ZnF) nanoparticles

The (Ax-CA-ZnF) conjugates were created by reacting CA-ZnF with an excess of Ax in the aqueous phase. The carboxylic acid groups on the surface of CA-ZnF NPs electrostatically interacted with the amino groups in AX. In this procedure, 50 μL of an aqueous dispersion of CA-ZnF NPs (2 mg/mL) was added to 1 mL of Ax solution (10 mg/mL in DMSO) and gently shaken at room temperature for 2 min^22^. Following the formation of the drug-nanoparticle conjugates, they were isolated from the unbound drug by magnetic separation and subsequent washing in an aqueous medium.

### Characterization of magnetic nanocomposites

The synthesized MNPs and coated MNPs, and conjugated MNPs were characterized at the central laboratories of the Galala University, the Military Technical College, Egyptian Armed Forces, using various techniques and instruments. The UV–vis spectra of the synthesized magnetic nanocomposites were analyzed in the 200–800 nm wavelength range using a UV–vis spectrophotometer (Agilent Technologies Cary 60 UV–vis). Under ambient circumstances, the samples were diluted and dispersed in double-distilled water.

The surface functionality and functional group composition of MNPs, coated MNPs, and conjugated MNPs were investigated by an FTIR 4700 spectrometer (Jasco, Tokyo, Japan) fitted with a Peltier stabilizer DLaTGS detector. Pellets of potassium bromide (KBr) were used to create specimens. With an average of 32 scans and a resolution of 4 cm^−1^, the FTIR spectra were acquired in the frequency range of 400–4000 cm^−1^.

The XRD-6000, Shimadzu equipment, SSI, Japan, was used to perform the X-ray diffraction patterns. The radiation source utilized was CuKα (40 kV, 40 mA), and the graphite monochromator was a secondary beam. A 2-theta (2θ) range of 10–80° with 0.02° increments and a 2 s counting time per step were among the patterns seen.

Equation 1 was used to calculate the average crystallite size using the Williamson-Hall (W–H) method^24^:

1 $$\beta {\text{cos}}\theta = k\lambda /D_{{(W{-}H)}} + { 4}\varepsilon {\text{sin}}\theta$$

where β is the full-width at half maximum, θ is the Bragg angle, k is a constant, λ is the X-ray wavelength, D (W–H) is the average crystallite size, and ε indicates the strain inside the samples.

A High-Resolution Transmission Electron Microscope was used to assess the size and shape of the produced magnetic nanoparticles (HRTEM, JEM2100, Jeol, Japan). A scanning electron microscope was used to analyze the grain size and surface morphology (SEM, ZEISS, EVO-MA10, Germany).

### Antimicrobial and antibiofilm activities of magnetic nanocomposites

#### Antimicrobial activity measurements

The agar-disc diffusion method was used to assess the synthetic samples’ antibacterial activity (10.0 μg/ml)^25^. The bacterial inoculums were standardized to 0.5 McFarland (1–3) × 10⁸ CFU/ml, with absorbance measured at 600 nm using a UV–Vis spectrophotometer^24^. After 24 h of incubation, the zone of inhibition (ZOI) was measured to determine the tested bacterial strains’ growth inhibition^22^. Conventional antibiotic discs, specifically Amoxicillin (AX; 25.0 μg/ml; 6.0 mm diameter), were used as a reference to evaluate the efficacy of the magnetic NPs.

#### Minimal inhibitory concentration (MIC) assay

As detailed in Sect. 2.4.1., the antimicrobial activity of the synthesized (ZnF-CA-AX) nanocomposite (10.0 μg/ml) was evaluated using the agar-disc diffusion method. The study assessed the inhibitory effects of synthesized nanoparticles on both *E. coli* (ATCC 25,922) as a model of gram-negative bacteria and *S. aureus* (ATCC 25,923) as a model for gram-positive bacteria. To assess the efficacy of the magnetic nanocomposites, conventional antibiotic discs (AX; 25.0 μg/ml; 6.0 mm diameter) were used. Using Luria–Bertani (LB) medium and the serial dilution method, the samples’ minimum inhibitory concentrations (MIC) were calculated to find the concentration with the strongest antibacterial activity^26^. The test’s positive control was the bacteria being studied, and the negative control was the medium broth containing ZnF-CA-AX NPs at an initial concentration of 10.0 μg/ml. Following a 24-h incubation period at 37.0 ± 0.5 °C, the MIC was determined^22^. As stated in the first antimicrobial screening, inoculums were prepared^25^. The ELISA plate method was used to estimate the MIC, and the wavelength was fixed at 600 nm^24^. The lowest concentration of ZnF-CA-AX nanocomposite that prevented 99.0% of the studied bacteria’s growth was known as the minimum inhibitory concentration (MIC).

#### Antibiofilm activity of magnetic ZnF-CA-AX nanocomposite

A qualitative assessment of biofilm formation was conducted using the methodology described by Maksoud et al^24^. Biofilm development on the tube walls was visually assessed in both the presence and absence of ZnF-CA-AX nanocomposite. ZnF-CA-AX nanocomposite (10.0 μg/ml) was evaluated against susceptible bacteria and its antibiofilm efficacy was compared to a control sample. In tubes with five milliliters of nutritional broth, an aliquot of bacteria (0.5 McFarland, (1–3) × 10^8^ CFU/ml) was added, and the tubes were then incubated for twenty-four hours at 37 °C. Following incubation, Phosphate Buffer Saline (PBS, pH 7) was used to wash the tubes and dehydrate them, and the contents of the treatment and control tubes were disposed of. After 10 min of fixing the bacterial layers sticking to the tubes with 3.0% of (5 ml) sodium acetate, deionized water was used to cleanse the tubes. The biofilms were stained with 0.1% Crystal Violet (CV) for 15 min, and any leftover stain was removed by rinsing them with deionized water. Five milliliters of ethanol were added to remove the color. A noticeable discolored layer on the tube’s bottom and surface suggested that a biofilm had formed^27^ The O.D. of the stained bacterial biofilms was measured using a UV–Vis spectrophotometer set to 570.0 nm in order to quantify the biofilms. The following (Eq. 2)^22^ was used to determine the inhibition percentage:

2 $$\begin{gathered} Bacterial{ }biofilm{\text{ inhibition }}\left( {\text{\% }} \right)\; = { } \hfill \\ { }\frac{{O.D.{\text{of control }}sample - O.D.{\text{of treated sample }}}}{{O.D.of{ }control{ }sample}}{\text{ X }}100{ } \hfill \\ \end{gathered}$$

### Antioxidant Activity of magnetic ZnF-CA-AX NCs

The antioxidant activity was assessed using the 1,1-diphenyl-2-picrylhydrazyl (DPPH) technique. 20 mg of ZnF-CA-AX NCs were evenly dispersed within a glass vial that previously contained 1.3 mL of DPPH solution (containing 100 µmol/L in methanol)^28^. The DPPH radical gradually transforms from its distinctive violet color when dissolved in a solution to a colorless or mild yellow hue when ZnF-CA-AX NCs are present. This feature makes it possible to easily monitor and follow the reaction. For comparison testing nanoparticles, ascorbic acid was used as a positive control or standard, while DPPH in methanol—aside from the powder samples—acted as a negative control sample. ZnF-CA-AX NCs and the DPPH reagent interacted more readily at the surface when modest magnetic stirring was used. After centrifugation, a supernatant was obtained and collected at 15-min intervals for analysis using UV–VIS spectroscopy at 517 nm in comparison to a methanol blank. Each sample was analyzed three times^29^. Equation 3 was used to obtain the percentage of inhibition in comparison to the blank:

3 $${\text{DPPH radical scavenging \% }} = { }\left[ {\left( {{\text{A}}0{ }{-}{\text{ A}}1} \right)/{\text{A}}0} \right]{ } \times { }100 { }$$

where A0 represents the DPPH solution’s absorbance and A1 represents the sample’s absorbance at a certain moment in time.

## Results and discussion

### Characterization of magnetic nanoparticles

MNPs are among the most widely utilized examples of nanotechnology’s application in medical fields. For any medical application, understanding the structure, surface functionality, and magnetic properties of these particles is crucial for researchers to assess and comprehend how these properties impact the medical challenges they aim to address^30^. Characterization is an essential first step in understanding how the function relates to the intrinsic properties of these particles^31^.

**Fig. 2:**
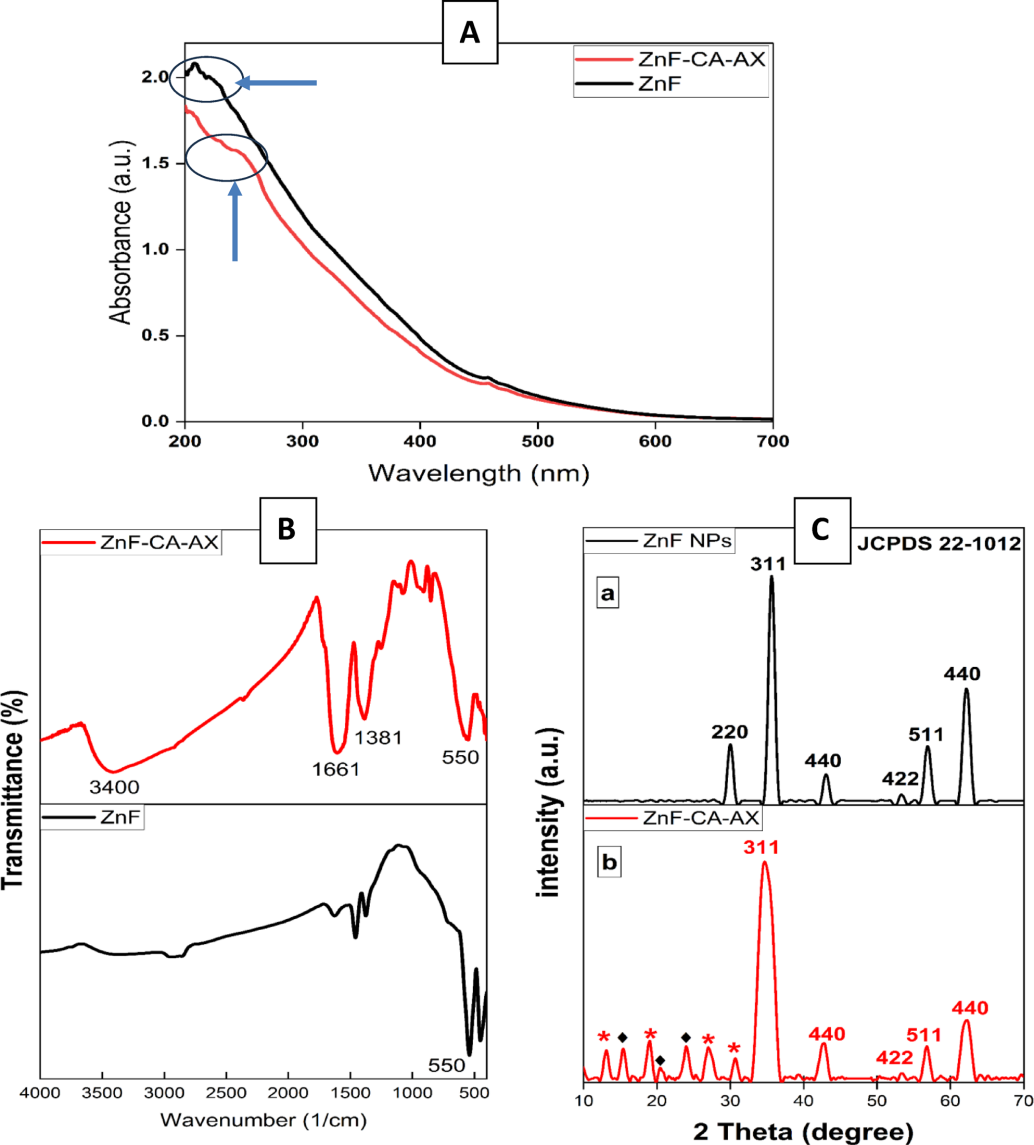
**(A)** UV spectrum of synthesized ZnF and ZnF-CA-AX NCs, the blue arrows and circles indicate the peaks of the two curves as indicated, (**B**) FTIR Analysis of Synthetic ZnF NPs, ZnF-CA NPs, and ZnF-CA-AX NCs, and (**C**) Synthesized ZnF NPs and ZnF-CA-AX NCs’ XRD patterns.

Throughout the visible spectrum, the synthesized magnetic nanoparticles (ZnF, ZnF-CA-AX) showed a distinct continuous peak absorption pattern, especially between 300 and 800 nm (Fig. 2A). This method was used to assess the optical characteristics and validate that the nanoparticles had been successfully functionalized. The naked zinc ferrite nanoparticles can be seen in the UV–visible range of 200–500 nm based on the absorption spectra^32^. According to our findings, ZnF NPs’ UV–vis spectrum peaks at 230 nm. After CA and AX conjugation with ZnF NPs, the peak of the UV–vis spectra of ZnF-CA-AX NCs moves to 270 nm. This shift is indicative of successful surface modification, as the interaction between the nanoparticles and the conjugated molecules alters the electronic environment of the ZnF NPs. This modification plays a critical role in enhancing the biological properties of the nanoparticles, particularly their antimicrobial and antibiofilm activities, by providing a more functionalized surface for interaction with bacterial cells.

The functional groups on the surface of the produced ZnF NPs were confirmed by FTIR analysis, which was also used to establish that CA and AX successfully conjugated onto the NPs (Fig. 2B). The typical sample’s Fe–O and Zn–O vibrations, which are indicative of the zinc ferrite structure, are the cause of the absorption peak seen at 550 cm^−1^ in ZnF. This confirms the crucial ZnF structure’s integrity. The large peak at 3400 cm^−1^ could be caused by the presence of structural hydroxyl groups and molecular water traces, which show surface hydration. The significant peak at 1381 cm^−1^ is caused by the C–H vibration band, while the peak at 1661 cm^−1^ is caused by the symmetric stretching of the C=O bond in the citric acid COOH group. These results demonstrate that a CA radical was attached to the ZnF NPs’ surface. Moreover, the bending vibration peaks in the CO=N–H around 900–1200 cm^−1^ suggests that AX and ZnF–CA are conjugated to create ZnF–CA–AX nanocomposites^22^, thus confirming the successful conjugation of AX with the NPs. Comparing the FTIR spectra of ZnF NPs and ZnF-CA-AX NCs before and after AX conjugation, no obvious alterations have been observed in the core ZnFe_2_O_4_ NPs, suggesting that the AX was conjugated and did not change the generated structure. Therefore, the conjugation of AX occurred without disrupting the nanoparticle’s properties, ensuring its structural integrity for biomedical applications. Moreover, functionalization and structural integrity were further supported by the UV–Vis spectroscopy results, which exhibited a distinct absorption peak at 230 nm for naked ZnF NPs, and at 270 nm after the conjugation of CA and AX. This shift clearly indicates changes in the electronic environment surrounding the nanoparticles due to the successful conjugation of the functional groups. The correlation between the FTIR and UV–Vis results highlight the effectiveness of the surface functionalization process and its implications for enhancing the NPs’ antibacterial properties.

To verify the crystallinity and phase purity of the produced ZnF NPs and their composites (ZnF-CA-AX NCs), XRD examination was performed. The diffraction patterns of both samples showing distinct intensity peaks at specific diffraction angles were shown in Fig. 2C. The angles of 30.12°, 35.54°, 43.13°, 56.89°, and 62.62° correspond to the reflection planes (220), (311), (400), (511), and (440), respectively, as referenced from standard ZnF diffraction data (JCPDS No. 22–1012). These well-defined peaks confirm that the NPs exhibit a cubic spinel structure and that there are no contaminants or significant oxidation, ensuring the structural integrity of the synthesized ZnF NPs. The average crystallite size at the strongest peak, the broadening diffraction peak at (311) was determined using the Scherrer’s Eq.^33^. The calculated crystallite sizes were 25 nm for ZnF NPs and 35 nm for ZnF-CA-AX nanocomposites, indicating a slight increase in size after AC and AX conjugation. In addition to the characteristic peaks of ZnF NPs, additional diffraction peaks (*) were observed at 12.9°, 18.7°, 26.9°, and 31.0°, corresponding to CA. This confirms the successful conjugation of CA with the Nps, further stabilizing the surface of the ZnF NPs. Similarly, the observed peaks (♦) at 15.3°, 20.7°, and 23.9°, corresponded to AX, confirming the conjugation of AX with the ZnF-CA NPs, (Fig. 2C), as shown by the UV–Vis spectrum and FTIR results. These combined results confirm that the CA and AX were successfully conjugated with the ZnF NPs while maintaining their structural and optical integrity. In conclusion, the XRD analysis not only confirmed the successful functionalization of the NPs with both CA and AX but also demonstrated that the conjugation process did not disrupt the crystallinity of the core ZnF structure.

#### Scanning electron microscopy (SEM) analysis

**Fig. 3:**
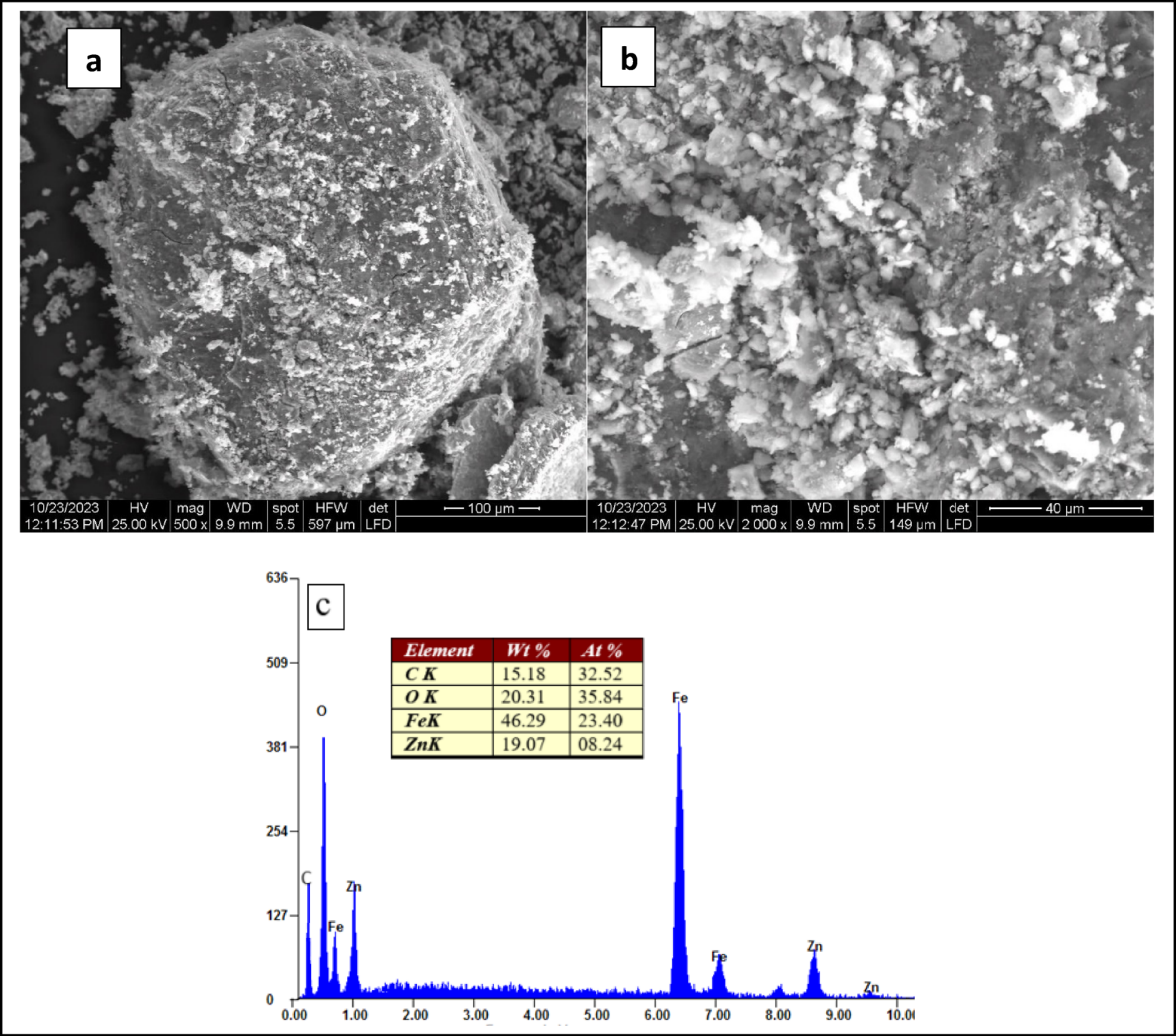
SEM images depicting surface morphology of (**a**) Zn Fe_2_O_4_ NPs (**b**) ZnF-CA-AX NPs (**c**) ZnF-CA-AX nanocomposite elemental analysis by EDX spectroscopy.

SEM analysis was used to evaluate magnetic NPs’ morphology, revealing important details about their physical characteristics and particle distribution on the surface structure (Fig. 3). While a uniform distribution of ZnF NPs was observed.

(Fig. 3a), the successful conjugation of ZnF NPs with AX and CA to create ZnF-CA-AX nanocomposites was revealed (Fig. 5b). The consistent, spherical shape shown in the SEM analysis confirms the efficacy of the synthesis procedure and NPs’ fundamental structure after the functionalization process.

Furthermore, ZnF-CA-AX NPs’ elemental composition was confirmed by Energy-Dispersive X-ray (EDX) analysis (Fig. 3c). The existence of CA and AX on the nanoparticle surface was confirmed by the appearance of carbon (C) and oxygen (O) peaks, as well as zinc (Zn) and iron (Fe) peaks, which further validates the effective functionalization. Since no contaminants were found, this elemental analysis further suggests good purity^22^. By demonstrating the presence of C and O in addition to Zn and Fe in the ZnF-CA-AX nanocomposites, the EDX analysis validates the successful functionalization of naked ZnF NPs with CA and AX.

#### Transmission electron microscopy (TEM) analysis

**Fig. 4:**
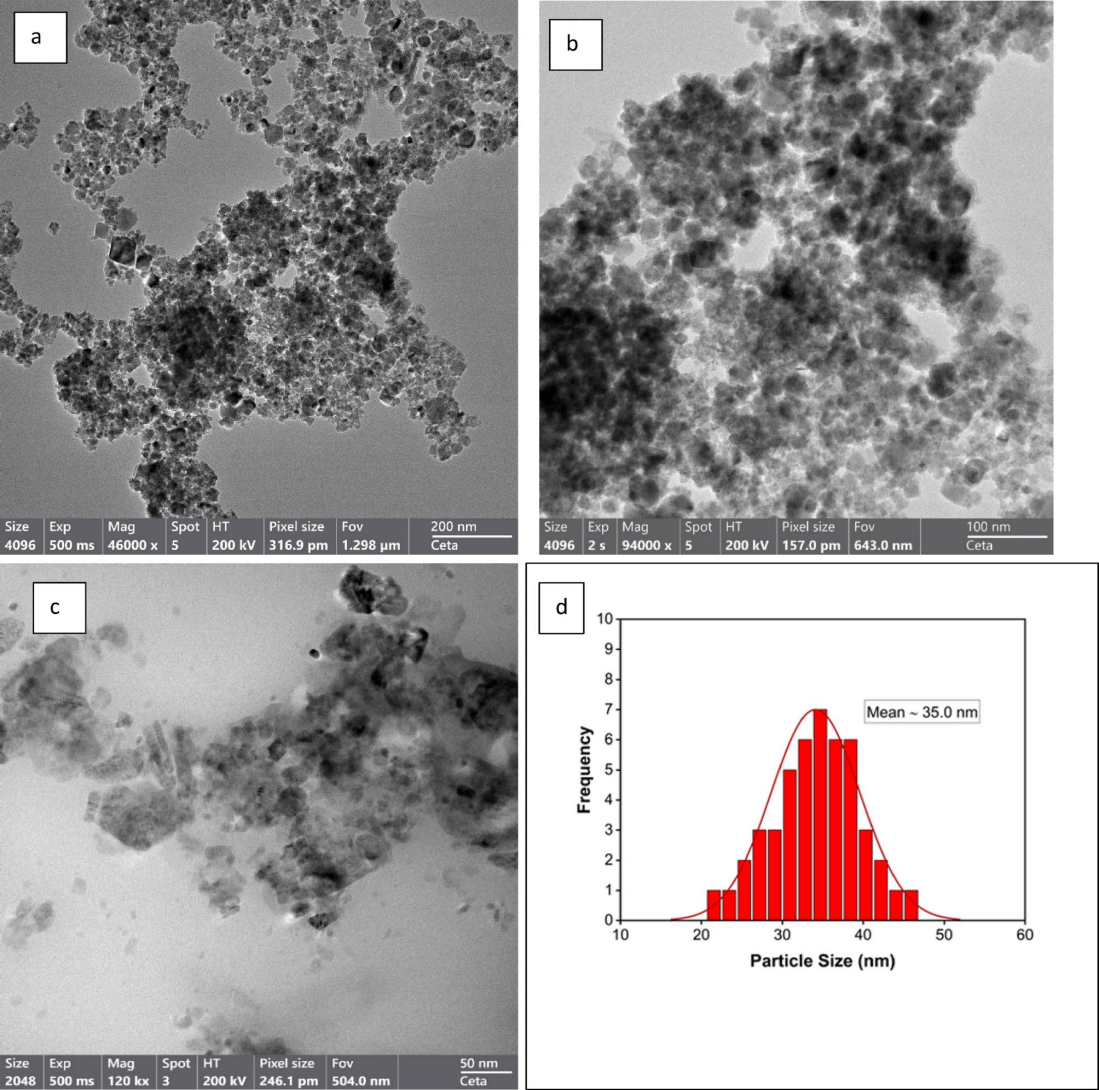
The HR-TEM images of (**a**) Naked ZnF NPs (**b**) ZnF-CA NPs (**c**) ZnF-CA-AX NCs, and (**d**) Particle size distribution of ZnF-CA-AX NCs.

High-resolution images from TEM allow for a thorough analysis of nanostructures, which is essential to understand their possible uses in biomedical domains. As shown in Fig. 4a, the naked ZnF NPs were remarkably tiny and semi-spherical in shape. The average ZnF particle size was 25.0 nm, with observed sizes ranging from 15.0 to 35.0 nm. The benefit of this small size is that it raises the surface area-to-volume ratio, which improves the reactivity and interactions of the NPs with the biological systems. Conversely, ZnF-CA NPs often have a smooth surface, a spherical form, and a modest size range of 20.0 to 40.0 nm, with an average diameter of 30.0 nm (Fig. 4b). The inclusion of CA affects shape and size distribution, which means good functionalization. TEM analysis of ZnF-CA-AX NCs showed spherical particles with a consistent size distribution (Fig. 4c). The histogram of the size of the ZnF-CA-AX NCs is given in Fig. 4d.

These particles’ average diameter of 35.0 nm was larger than that of the synthesized naked ZnF nanoparticles due to the conjugation with CA and AX. Thus, this XRD analysis corroborates the previous findings on average crystallite sizes, indicating that the increase in size was associated with the conjugation of CA and AX. In summary, the TEM analysis not only confirmed the spherical morphology and size distributions of the synthesized nanoparticles but also confirmed the successful functionalization as indicated by previous results.

### Antibacterial and antibiofilm activities of the magnetic nanocomposites

#### Antibacterial activity and minimum inhibitory concentration (MIC) assay

**Fig. 5:**
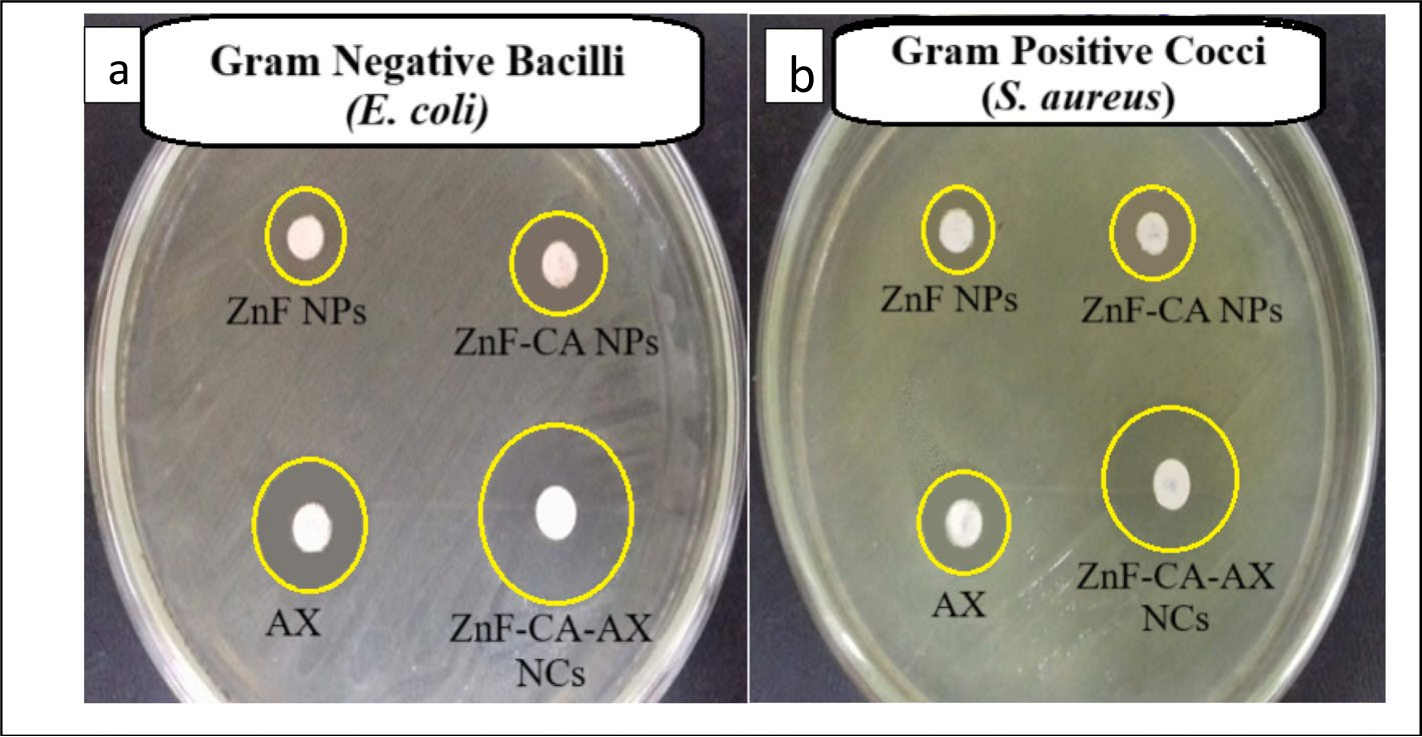
The zone of inhibition (ZOI) for the antimicrobial activity of synthesized nanoparticles against (**a**) Gram-negative (*E. coli*) and (**b**) Gram-positive (*S. aureus*) bacteria. DMSO is utilized as a negative control, and the common antibiotic Amoxicillin (AX) is employed as a positive control.

The synthesized MNPs’ antibacterial activity against one strain of gram-positive bacteria (*S. aureus*) and one strain of gram-negative bacteria (*E. coli*) was evaluated using the agar well diffusion technique. Each bacterial strain’s ZOI was evaluated in relation to the negative control (DMSO) and the positive control (AX). The lack of microbial growth in the surrounding area of NPs may be interpreted as the NPs’ capacity to inhibit or suppress microbial growth. According to the findings of a screening experiment employing the disc agar diffusion method, the ZnF-CA-AX nanocomposite exhibited qualitative antibacterial activity against the examined bacteria (Fig. 5).

According to the in-vitro ZOI data displayed in Table 2, ZnF-CA-AX NCs showed strong antibacterial activity against *S. aureus* and *E. coli*, with corresponding ZOIs of 17.0 and 20.0 mm. The much higher antibacterial activity of ZnF-CA-AX NCs in comparison to ZnF-CA and ZnF NPs suggests that AX and ZnF-CA NPs may have synergistic effects.

**Table 2:** Antimicrobial activity of ZnF NPs, ZnF-CA NPs, ZnF-CA-AX NCs, and Amoxicillin against *S. aureus* and *E. coli*.

Bacterial strains	ZOI of ZnF NPs (10.0 µg/ml) (mm)	ZOI of ZnF-CA NPs (10.0 µg/ml) (mm)	ZOI of ZnF-CA-AX (10.0 µg/ml) (mm)	AX (25.0 µg/disc)	MIC of ZnF-CA-AX NPs (µg/ml)	OD (Control sample)	OD (Treated)	Antibiofilm (%)
*S. aureus*	9.0 ± 0.23	11.0 ± 0.62	17.0 ± 0.35	13.0 ± 0.40	2.50	1.95	0.229	88.4
*E. coli*	10.0 ± 0.40	13.0 ± 0.70	20.0 ± 0.54	15.0 ± 0.23	1.25	1.83	0.117	93.7

Results are presented as zone of inhibition (ZOI) in millimeters (mm), minimum inhibitory concentration (MIC) in micrograms per milliliter (µg/mL), and antibiofilm activity (%) for ZnF-CA-AX NCs.

The antibacterial qualities of a nanocomposite cannot be determined solely by size; other crucial factors such as elemental composition, purity, surface area, and form must be carefully considered^34^. ZnF-CA-AX NCs have two advantages that facilitate their interaction with biological organisms such as bacteria and yeast: a high surface-to-volume ratio and a nanoscale structure. The minimum inhibitory doses (MIC) of ZnF-CA-AX NCs, ZnF-CA NPs, and ZnF NPs against the different bacterial strains ranged from 20.0 to 0.312 μg/mL (Table 2).

The results reported in Table 3 confirmed the antimicrobial efficacy of the various nanomaterials tested against multiple strains of *E. coli* and *S. aureus*, ranging from standard laboratory strains to pathogenic and multidrug-resistant (MDR) variants. The distinction between these strains is critical to understanding the effectiveness of the nanocomposites in real-world applications, especially against MDR pathogens, which present significant challenges in clinical treatments. The results highlight the MIC and ZOI for each material, offering insights into the potential of these nanocomposites to limit the bacterial pathogen growth. Notably, Fe₃O₄/Ag, MgFe₂O₄ NPs, and CA-MgFe₂O₄ NPs exhibit significant antimicrobial activity, demonstrating promising efficacy. In another study by Zhang et al. clarithromycin-loaded nanoparticles cleared off the population of *H. pylori* in both *in*-*vitro* and *in*-*vivo* conditions. The antibacterial effect of clarithromycin-loaded nanoparticles was found to be significantly higher than free clarithromycin antibiotics^46^. Additionally, combinations such as Fe₃O₄/Ag with Sulfamethoxazole (SMX) and Trimethoprim (TMP) further enhance their antibacterial effects, reflecting synergistic benefits. Other materials, such as ZnO and its combination with Ciprofloxacin, show an increased ZOI, indicating enhanced antibacterial potency.

**Table 3:** Antimicrobial efficacy of different nanomaterials against *S. aureus* and *E. coli* bacteria.

Nanocomposite	Method of synthesis	Particles size (nm)	Pathogen	MIC (μg/mL)	ZOI (mm)	Refs.
Fe_3_O_4_/Ag	Chemical reduction method	33.2	*E.coli*	10	N/A	^35^
			*S.aureus*	10		
Sulfamethoxazole (SMX)		–	*E.coli*	12		
			*S.aureus*	12		
Fe_3_O_4_/Ag/SMX		–	*E.coli*	4		
			*S.aureus*	4		
Trimethoprim (TMP)		–	*E.coli*	9		
			*S.aureus*	9		
Fe_3_O_4_/Ag/TMP		–	*E.coli*	4		
			*S.aureus*	4		
MgFe_2_O_4_ NPs	Co-precipitation method	–	*E.coli*	10	11.0 ± 0.26	^36^
			*S. aureus*	10	12.0 ± 0.40	
Gentamycin		–	*E.coli*	N/A	15.0 ± 0.30	
			*S. aureus*		26.0 ± 0.52	
CA-MgFe_2_O_4_ NPs		–	*E.coli*	2.5	16.0 ± 0.50	
			*S.aureus*	1.25	20.0 ± 0.49	
AgNPs	Chemical reduction method	35.50	*E.coli*	N/A	20 ± 1.5	^37^
Ag-AX NPs		–	*E.coli*	N/A	40 ± 1.2	
Amoxicillin (AX)	Green synthesis method	–	*E.coli*	N/A	19 ± 0.64	^38^
			*S.aureus*	N/A	25 ± 0.41	
GNP-AX		33.9 ± 14	*E.coli*	N/A	31 ± 0.99	
			*S.aureus*	N/A	30 ± 0.75	
Streptomycin	Co-precipitation method	–	*E.coli*	N/A	19.7 ± 0.04	^39^
			*S.aureus*	N/A	22.5 ± 0.02	
Zinc ferrite NPs		10	*E.coli*	N/A	14.6 ± 0.08	
			*S.aureus*	N/A	22.2 ± 0.05	
(Amoxicillin/Clavulanic acid; 20/10 μg/mL)	Sol–gel method	–	*E.coli*	N/A	17.0 ± 0.57	^40^
		–	*S.aureus*		16.0 ± 1.00	
CFO		19.42	*E.coli*		9.0 ± 0.00	
			*S.aureus*		8.0 ± 0.57	
CCFO		33.71	*E.coli*		12.0 ± 0.57	
			*S.aureus*		10.0 ± 0.28	
ZCFO		13.04	*E.coli*		11.0 ± 0.57	
			*S.aureus*		13.0 ± 0.57	
MCFO		30.96	*E.coli*		12.0 ± 0.28	
			*S.aureus*		9.0 ± 0.50	
Ciprofloxacin	precipitation method	–	*E.coli*	N/A	4	^41^
ZnO		24.84	*E.coli*		12	
ZnO- Ciprofloxacin		–	*E.coli*		32	
Levofloxacin	Chemical reduction method	–	*S.aureus*	2	N/A	^42^
Levofloxacin–AgNPs			*S.aureus*	0.5	N/A	
Streptomycin	Biological synthesis method	–	*E.coli*	N/A	25	^43^
			*S.aureus*		21	
SNP		30	*E.coli*	N/A	13	
			*S.aureus*		15	
Streptomycin-SNP		–	*E.coli*	N/A	30	
			*S.aureus*		23	
Tetracycline (Tet)	Co-precipitation method	–	*E.coli*	8	N/A	^44^
			*S.aureus*	8		
Tet-met-NiFe_2_O_4_		27	*E.coli*	1		
			*S.aureus*	4		
Streptomycin	Green synthesis method	–	*S.aureus*	N/A	Nil	^45^
Streptomycin-ZnO		4.77	*S.aureus*	N/A	11 ± 0.5	
Streptomycin-FeO		15.3	*S.aureus*	N/A	8 ± 0.5	
ZnFe_2_O_4_	Co-precipitation method	25	*E.coli*	NA	10.0 ± 0.40	Our study
			*S.aureus*		9.0 ± 0.23	
ZnF-CA		–	*E.coli*	NA	13.0 ± 0.70	
			*S.aureus*		11.0 ± 0.62	
ZnF-CA-AX		35	*E.coli*	1.25	20.0 ± 0.54	
			*S.aureus*	2.5	17.0 ± 0.35	
Amoxicillin (AX)		–	*E.coli*	NA	15.0 ± 0.23	
			*S.aureus*		13.0 ± 0.40	

N/A indicates that the zone of inhibition (ZOI) was not measured, as the experiment was not conducted for this condition., Nil indicates that no zone of inhibition (ZOI) was observed.

Moreover, the results reported in Table 3 indicate that ZnF-CA-AX shows a significant increase in ZOI against *E. coli* and *S. aureus*. The absence of measurable ZOI or MIC in some cases (marked as N/A or Nil) suggests either no testing or lack of efficacy under certain conditions, further emphasizing the need to assess material performance under different experimental settings.

This data collectively underscores the potential of nanoparticles and antibiotic-nanoparticle hybrids as potent antimicrobial agents against antibiotic-resistant bacteria.

#### Antibiofilm activity

**Fig. 6:**
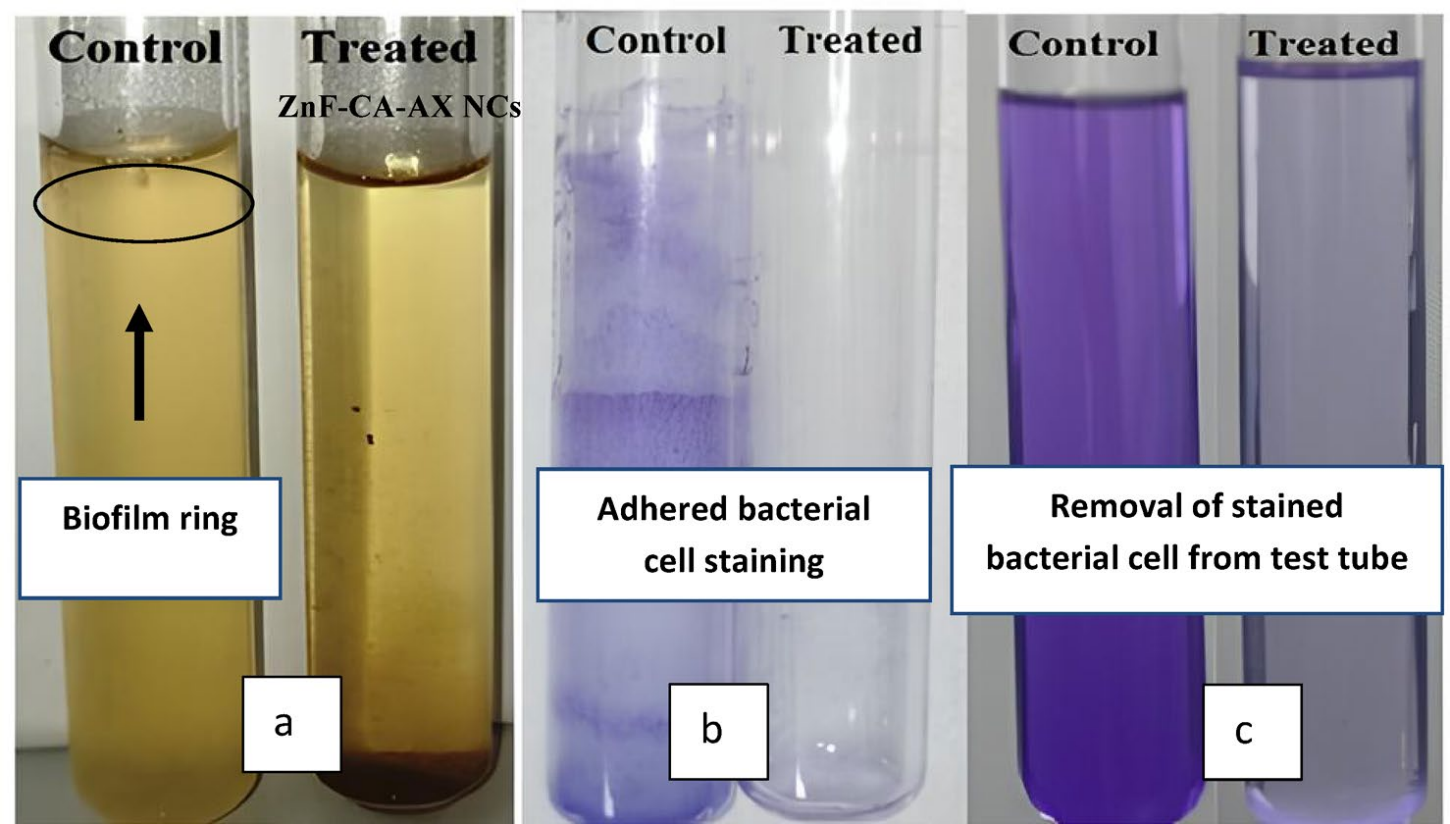
Test tube technique evaluation of ZnF-CA-AX NCs’ antibiofilm efficacy against *E. coli*. The following is the order of actions: (**a**) In contrast to decreased bacterial growth after treatment with the NCs, bacterial cell proliferation and biofilm formation (shown by rings) were seen in the absence of ZnF-CA-AX NC treatment. (**b**) Using crystal violet to see the germs that have stuck to the surface. (**c**) The attached bacterial cells are dissolved and removed using ethanol, allowing for a semi-quantitative evaluation of the inhibition of biofilm formation (%).

Many microbes that produce exopolysaccharides have been shown to create biofilms^47,48^. The antibiofilm activity of ZnF-CA-AX NCs against *E. coli* was analyzed using a test tube approach (Fig. 6). *E. coli* formed a concentrated whitish-yellow film that covered the whole air–liquid interface in the absence of ZnF-CA-AX NCs. The film exhibited a circular blue structure and demonstrated great adhesion to the test tubes’ inner walls after Crystal Violet staining. A blue-colored suspension was produced by dissolving the stained ring in 99.0% ethanol. The optical density was measured at 570.0 nm^24^. On the contrary, test tubes that were injected with *E. coli* and then treated with ZnF-CA-AX NCs at a dose of 10.0 µg/ml showed a significant inhibitory effect (Table 2).

The greatest degree of inhibition against *E. coli* (93.7%) and *S. aureus* (88.4%) was observed at a dose of 10.0 µg/ml of the ZnF-CA-AX NCs. The observed variation in the inhibitory percentage may be due to a variety of factors, including antibacterial properties, biosorption capacity, physical characteristics, invasive potential, and special chemical features that regulate the interaction between the nanomaterials and the biofilms.

#### Proposed mechanism of antimicrobial activity of synthesized ZnF-CA-AX NCs

**Fig. 7:**
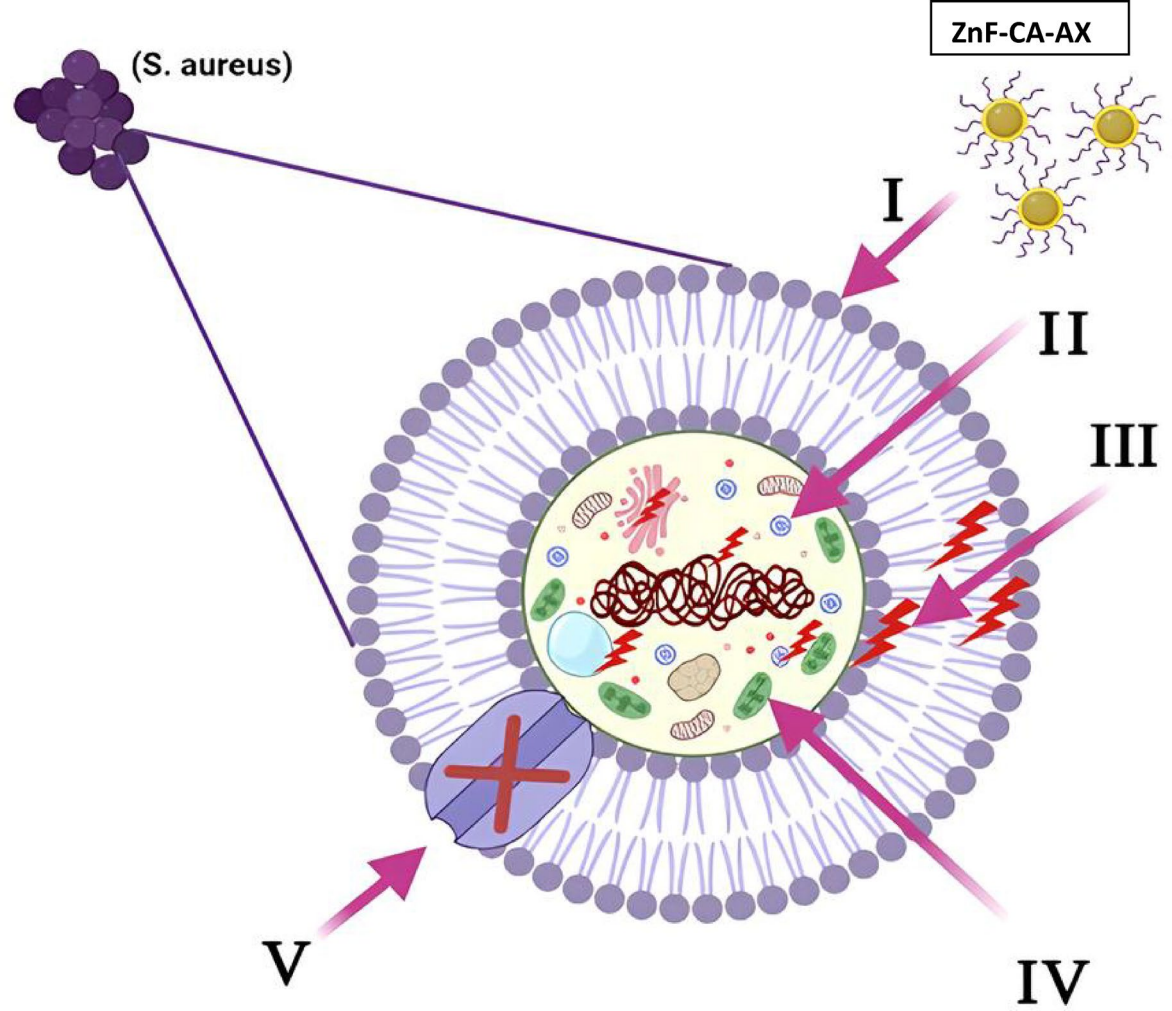
Schematic representation of the five key antibacterial mechanisms exhibited by ZnF-CA-AX nanocomposites (NCs): (I) ZnF-CA-AX NCs adhere to the microbial cell surface, causing membrane disruption and altering transport activities. (II) NCs penetrate microbial cells, interacting with intracellular biomolecules and affecting cellular functions. (III) ZnF-CA-AX NCs promote the generation of reactive oxygen species (ROS), leading to oxidative damage within the cell. (IV) The NCs cause programmed cell death by interfering with cellular signaling pathways. (V) ZnF-CA-AX NCs further disrupt cellular functions by interfering with ion transport across the microbial cell membrane.

Magnetic NPs have been shown to be excellent antibacterial agents, and metals’ detrimental effects can be assessed in both bacterial and eukaryotic species^49^. However, few publications on magnetic nanocomposites exist, and it is still unknown what chemical mechanism exactly makes them antibacterial^50^. Reactive oxygen species (ROS) generated as molecules, ions, and radicals through various chemical and biological processes are characterized by relatively short half-lives ranging from nanoseconds to hours^51^. ROS generation is one of the principal mechanisms by which metal-based nanocomposites exert their antimicrobial effects. ROS such as hydroxyl radicals (•OH), superoxide anions (O₂•^−^), and singlet oxygen (^1^O₂) can disrupt bacterial membranes, damage proteins, and induce DNA fragmentation, ultimately leading to cell death. The ZnF-CA-AX nanocomposite developed in this study is expected to promote ROS generation, which may synergize with Amoxicillin’s antibacterial action to enhance efficacy against resistant and biofilm-forming strains. Previous studies have demonstrated the significant role of ROS in bacterial inactivation using metal oxide nanoparticles, supporting this proposed mechanism^51,52^. Although direct measurement of ROS was not conducted in this study, the findings aligned with previous reports that demonstrated the role of ROS in the antimicrobial activity of similar nanomaterials. Recent studies have used DCFH-DA fluorescence assays, ESR spectroscopy, and colorimetric methods such as NBT reduction to confirm ROS generation in comparable systems^53^. Further studies will focus on confirming ROS production to validate this proposed mechanism and further elucidate the interaction between the nanocomposite and bacterial cells. The synthesized magnetic NPs have a synergistic effect in breaking down cellular structures and preventing the proliferation of bacteria^49^ (Fig. 7). Kalita et al. explains that antibiotic-NPs conjugate target multiple sites within bacterial cells rather than merely interacting with or penetrating the cell wall. Once inside, these conjugates disrupt vital regulatory processes, deactivate essential bioactive proteins, and interfere with sulfur-phosphorus interactions within nucleic acids, ultimately inhibiting protein synthesis and halting bacterial growth^38^.

### Antioxidant activity

**Fig. 8:**
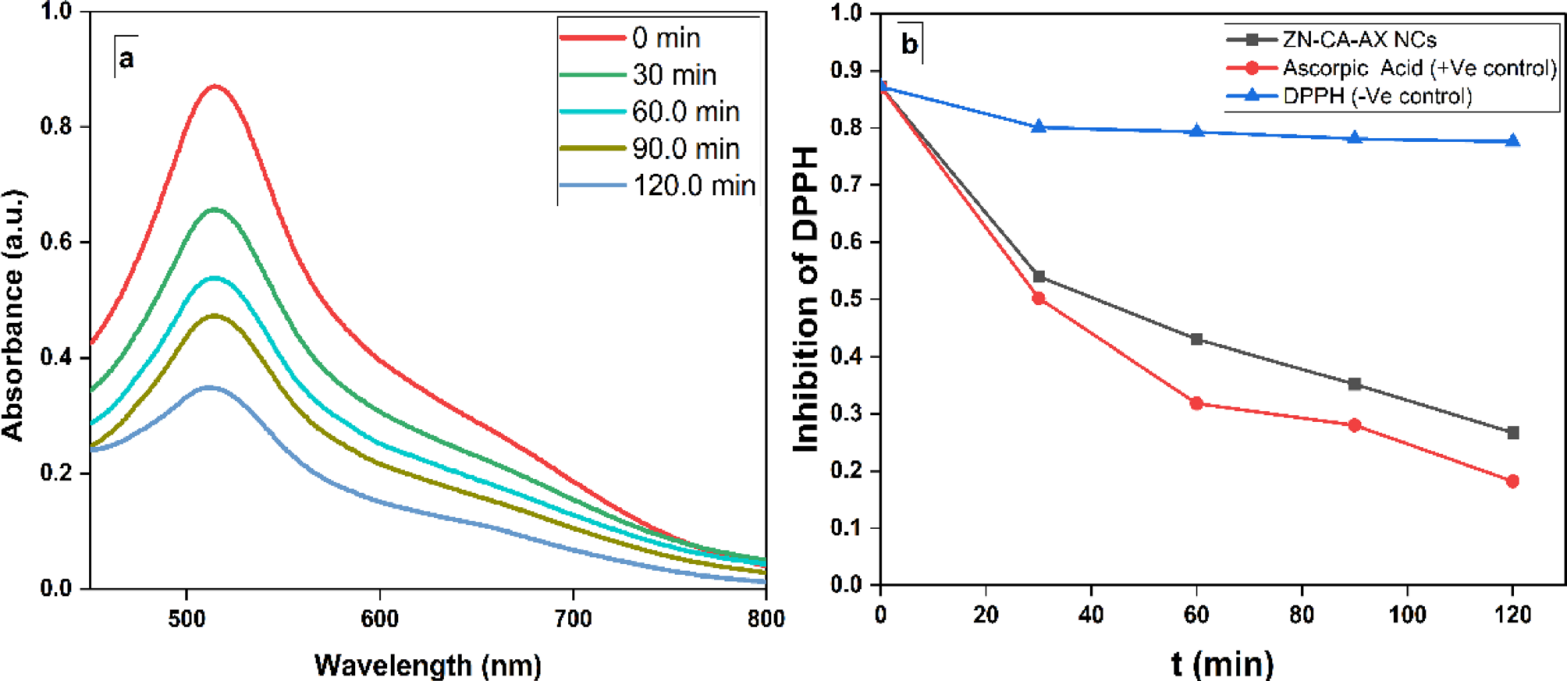
**(a)** DPPH’s UV–Vis spectrum at various concentrations throughout time (**b**) ZnF-CA-AX NCs’ capacity to scavenge DPPH.

It was observed that the DPPH solution’s color gradually shifts from deep violet to pale yellow when ZnF-CA-AX NCs are present. The UV–visible spectra of DPPH in the presence of ZnF-CA-AX NCs at different time intervals was analyzed (Fig. 8a). The decrease in absorbance at 517 nm was used to measure the NPs’ DPPH scavenging capability^54,55^. The consistent drop in intensity at 517 nm over time demonstrated the ZnF-CA-AX NCs’ ability to scavenge free radicals (Fig. 8b).

These findings demonstrated that, during a 120-min period, the ZnF-CA-AX NCs’ DPPH scavenging activity was 69.0%, compared to 80.0% for the ascorbic acid (positive control).

## Conclusion

This work aims to try to find potential solutions to the antibiotic resistance problem via the use of functionalized MNPs. Here, we focused on the preparation of ZnF nanoparticles through an aqueous co-precipitation method and coating those with AC for functionalization. The primary characteristics of magnetic particles that are heavily utilized in medical applications are their high-level accumulation in the target tissue or organ, biocompatibility, injectability, and lack of toxicity. Next, we focused on the evaluation of the antimicrobial activity against gram-positive and gram-negative bacteria. The synthesized ZnF-CA-AX demonstrated enhanced antibacterial activity against *S. aureus* (17.0 ± 0.35 mm ZOI), and *E. coli* (20.0 ± 0.54 mm ZOI) respectively; the developed nanocomposite demonstrated advanced anti-film activity with an inhibition percentage of 93.7% against *E. coli* followed by 88.4% against *S. aureus*. Additionally, ZnF-CA-AX NC demonstrated antioxidant activity against DPPH with 69.0%, compared to 80.0% for the ascorbic acid as positive control. Collectively, these results suggest that ZnF-CA-AX NCs hold potential for biomedical applications due to their enhanced antimicrobial activity. However, their use should be limited for specific purposes, as their toxicity needs further evaluation. Potential applications include antimicrobial coatings for operating room walls, face masks, cosmetics, and wound dressings, where they could serve as a promising antimicrobial agent.

## Data Availability

The datasets used and analyzed during the current study are available from the corresponding author on reasonable request.
